# Brinker regulates reciprocal outcomes of BMP signal between stem cells and differentiating cells

**DOI:** 10.1101/2025.09.14.676154

**Published:** 2025-09-16

**Authors:** Samaneh Poursaeid, Jeffrey P Gamer, Mayu Inaba

**Affiliations:** 1. Department of Cell Biology, University of Connecticut Health Center, Farmington, Connecticut, United States of America

**Keywords:** Germline stem cells, niche, Asymmetric division, Bone Morphogenetic Protein, Brinker

## Abstract

*Drosophila* male germline stem cells (GSCs) reside at the testis tip, surrounding a cluster of niche cells known as the hub. Bone Morphogenetic Protein (BMP) ligands secreted from the hub exert both contact-dependent and -independent effects. In close proximity to the niche, BMP signaling maintains stem cells by suppressing transcription of the key differentiation factor Bag of Marbles (Bam). In contrast, the diffusible fraction of BMP promotes differentiation of cells by activating bam. How a single signaling pathway produces such opposing outcomes has remained unclear. Here, we show that the diffusible BMP fraction induces bam transcription by repressing the transcriptional repressor Brinker (Brk). We further found that *brk* mRNA displays a highly heterogeneous expression pattern within interconnected spermatogonial cysts, suggesting that Brk may prime cell fate in a subset of transit-amplifying cells, helping to preserve a population poised for dedifferentiation while maintaining other cells for differentiation. Our findings propose a model in which a single niche-derived factor modulates reciprocal outcomes inside versus outside the niche, which is essential for the tissue homeostasis. Given the broad use of BMP signaling across stem cell niches, this mechanism may represent a general strategy to ensure correct balance between self-renewal and differentiation of stem cells.

## Introduction

Many adult stem cells divide asymmetrically to produce two daughters with distinct fates, one retains stem-cell identity, while the other initiates differentiation. Although asymmetric division is an efficient strategy for stem-cell maintenance, stem cells have finite lifespans, and lost cells can be replenished either through symmetric self-renewal divisions or by dedifferentiation^1–6^. We previously demonstrated that dedifferentiation, where partially differentiated cells revert to stem-cell identity, is the predominant mechanism for maintaining germline stem cells (GSCs) in the *Drosophila* testicular niche^1,7,8^. Despite its importance, the molecular and cellular mechanisms governing dedifferentiation remain poorly understood.

**Figure 1.**
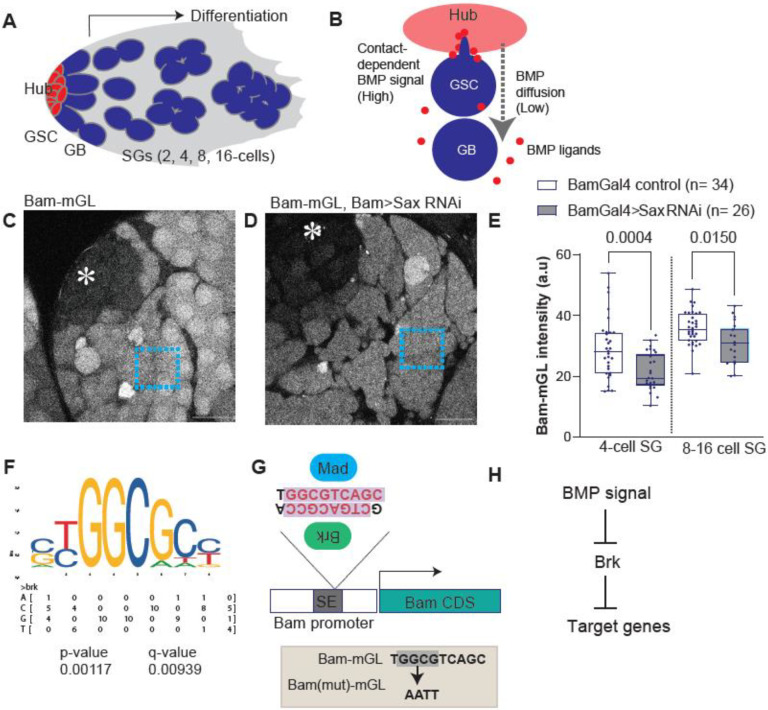
Bam promoter contains putative Brinker (Brk) binding site (A) The diagram shows anatomy of *Drosophila* testicular niche. GSC: Germline stem cell, GB: Gonialblast, SGs: Spermatogonia. (B) Asymmetric division of a male GSC. BMP signal mediates distinct outcomes between GSC and GB/SG populations via contact dependent and independent mechanisms, respectively. (C, D) Representative images of Bam transcriptional reporter (Bam-mGL) of indicated genotypes. Square regions in the images indicate examples of measured area on 8–16 SG stage. Asterisks indicate the hub. Scale bars indicate 20μm. (E) Intensity quantification of Bam-mGL reporter in 4- or 8–16 cell SGs. p-values were calculated by Šídák’s multiple comparisons tests and provided on each graph. n indicates the number of scored cysts. (F) Brk binding motif obtained from JASPER (matrix profile; MA0213.1) and nucleotide frequency matrix. P-value and q-value were obtained for bam promoter sequence by motif scanning. (G) Structure of bam promoter. Previously identified Mad binding sites are potentially recognized by Brk on its antisense strand. Lower box shows mutated nucleotides for Bam(mut)-mGL reporter construct. All reporters contain bam promoter from position −198 relative to TSS to endogenous start codon of bam gene. (H) Suggested relationships between BMP signal and Brk.

The *Drosophila* testis provides a simple and powerful model to study stem-cell regulation in the niche. Each testis harbors 8–10 GSCs that directly contact a central cluster of niche cells, the hub (Figure 1A)^9,10^. Upon GSC division, one daughter remains attached to the hub and retains stem-cell identity, while the other initiates differentiation program, four synchronous transit-amplifying divisions to form a 16-cell spermatogonial (SG) cyst (Figure 1A)^4,11–14^. The Bone Morphogenetic Protein (BMP) ligand, Decapentaplegic (Dpp), secreted from the hub, is essential for GSC maintenance by repressing transcription of the key differentiation factor Bam^15–19^. When a daughter cell loses hub contact, bam expression is activated, allowing entry into the differentiation program^15–19^.

For nearly two decades, Dpp was thought to act strictly in a short range, restricted to GSCs in direct hub contact, ensuring that signaling is limited to stem cells and excluded from differentiating daughters. However, our previous work revealed that a diffusible fraction of Dpp does exist outside the niche and exerts the opposite effect on bam expression. While niche-bound Dpp represses bam in GSCs, diffusible Dpp promotes bam expression in differentiating cells, thereby preventing excessive dedifferentiation^20^. These opposing outcomes likely arise from different concentration of Mothers against Dpp (Mad), the downstream transcription factor of BMP pathway (Figure 1B)^20^. However, the molecular basis for this dual action remains unclear.

In this study, we investigate the role of another transcription factor, Brinker (Brk) as a downstream factor of diffusible niche-derived BMP signaling. Our findings suggest that Brk mediates the opposing effects of BMP signaling on bam in GSCs and differentiating cells.

## Results

### Bam promoter contains putative Brinker (Brk) binding site

In *Drosophila* female germline stem cells (GSCs), bam transcription is repressed by niche-derived BMP signaling, similar to the case in the testis^21,22^. This repression occurs through a silencer element (SE) within the bam promoter, which is directly bound by the canonical BMP pathway effector, Mad and its partner Medea (Med)^21^. To study bam transcriptional regulation in the male germline, we used a previously established transcriptional reporter in which the bam core promoter (198-bp upstream of the transcription start site) drives expression of the fluorescent protein mGreenLantern (Bam-mGL)^20^. Consistent with endogenous bam expression, Bam-mGL is strongly activated beginning in the 4- to 8-cell spermatogonial (SG) stage, and remains high in later SGs (Figure 1C)^20^.

To ensure the role of BMP signal on the Bam promoter, we examined Bam-mGL activity under conditions of perturbed BMP pathway. In our previous study, we determined that the type I BMP receptor saxophone (Sax) specifically transduce the signal from diffusible fraction of BMP to differentiating SGs^20^. Consistent with the previous report, knockdown of Sax combined with the Bam-mGL reporter led to significantly weaker Bam-mGL intensity in SGs (Figure 1C–E).

These results suggest that BMP signaling modulates *bam* transcription through the SE in a location-dependent manner, such that suppressing *bam* transcription in GSCs while enhancing it in differentiating spermatogonia. This dual activity raises the possibility that multiple transcriptional regulators act on the bam promoter, and that the interplay between these factors downstream of BMP signaling determines the final transcriptional outcome.

A strong candidate for such a regulator is Brinker (Brk), a transcriptional repressor that is a well-established antagonist of BMP signaling across diverse developmental contexts^23,24^. Brk typically binds to promoter of BMP-responsive genes and represses their transcription, while BMP signaling itself represses *brk* expression, thereby permitting target gene activation^24^.

To test whether Brk may directly influence *bam* transcription, we performed motif-scanning analysis of the SE using FIMO^24–26^ with the Brk consensus binding sequence obtained from JASPAR^26^. Strikingly, this analysis revealed that the previously determined Mad-binding site within the SE overlaps with a potential Brk-binding site (Figure 1F, G)^22^. This observation suggested a unique relationship of Mad and Brk at the SE, whereby BMP signaling might indirectly activate *bam* expression by suppressing *brk* expression, as has been documented in other systems (Figure 1H)^27^.

### Brk suppresses bam transcription in SGs

**Figure 2.**
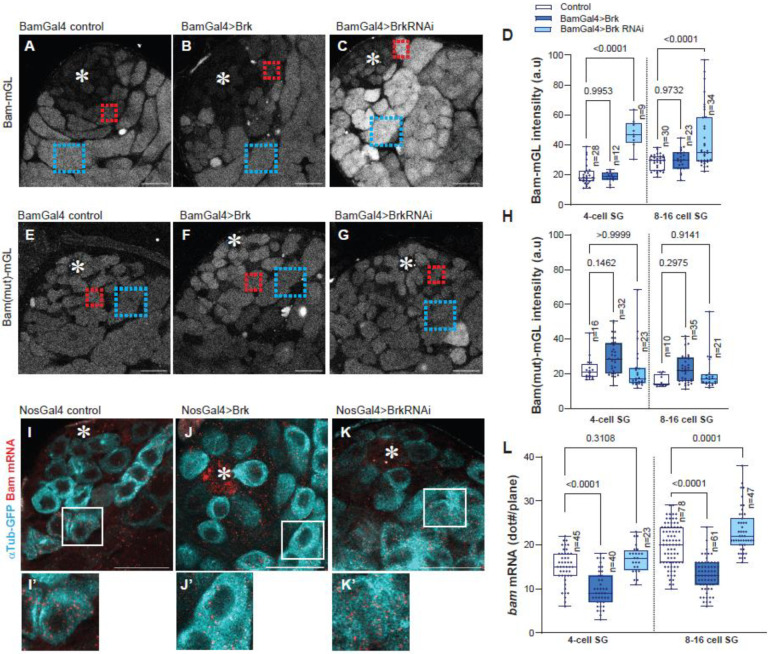
Brk suppresses bam transcription in SGs (A-C) Representative images of Bam transcriptional reporter (Bam-mGL) in indicated genotypes. (D) Intensity quantification of Bam-mGL reporter in 4- or 8–16 cell SGs for indicated genotypes. n indicates the number of scored cysts. (E-G) Representative images of mutated version of Bam transcriptional reporter, Bam(mut)-mGL, in indicated genotypes. (H) Intensity quantification of Bam(mut)-mGL reporter in 4- or 8–16 cell SGs for indicated genotypes. n indicates the number of scored cysts. Squared regions in the images (A-C, E-G) indicate examples of measured area on 4-cell SG stage (red) or 8–16-cell SG stage (blue). (I-K) Testis-tip images of bam mRNA FISH (red) for indicated genotypes. Germ cells are visualized by NosGal4>αTub-GFP (Cyan). Lower panels (I’-K’) show magnified images of squared area in I-K. (L) mRNA quantification from mid-plane of cells in indicated stages. mRNA molecules (smFISH dots/plane) were manually counted in the cells in indicated stages. n indicates the number of scored cells. p-values were calculated by Šídák’s multiple comparisons tests in all graphs and provided on each graph. Asterisks indicate the hub. All scale bars indicate 20μm.

Given the presence of a putative Brk-binding site within the bam promoter, we next asked whether Brk directly represses *bam* expression in SGs. To address this, we used the bam transcriptional reporter, Bam-mGL, in combination with perturbations of Brk levels specifically in the germline. Overexpression of Brk under the BamGal4 driver led to a marked increase in Bam-mGL intensity in SGs, whereas Brk knockdown caused a significant reduction of Bam-mGL signal in both 4-cell and 8–16 cell SGs compared with controls (Figure 2A–D). Importantly, these effects were abolished when the Bam(mut)-mGL reporter was used, in which the putative Brk-binding site was disrupted (Figure 2E–H). These results strongly suggest that Brk directly binds to the SE in the *bam* promoter to regulate reporter activity.

To validate these findings at the endogenous locus, we performed single-molecule fluorescent in situ hybridization (smFISH) against *bam* mRNA^28^. Consistent with the reporter assays, Brk knockdown significantly increased the number of *bam* mRNA molecules in SGs, whereas Brk overexpression led to a substantial reduction in *bam* transcripts (Figure 2I–L). Together, these results indicate that Brk suppresses excessive *bam* transcription in spermatogonia by directly acting on the SE, located within the *bam* promoter.

### BMP signal suppresses Brk expression in SGs

In early embryogenesis and wing imaginal disc development, Brk represses BMP-responsive genes, and its own transcription is negatively regulated by Dpp signaling through the Mad-Medea-Schnurri complex^29^. Similarly, in the *Drosophila* ovary, the expression of *brk* is suppressed by BMP signal^30^, raising the possibility that the same regulatory principle also applies in the testis.

**Figure 3.**
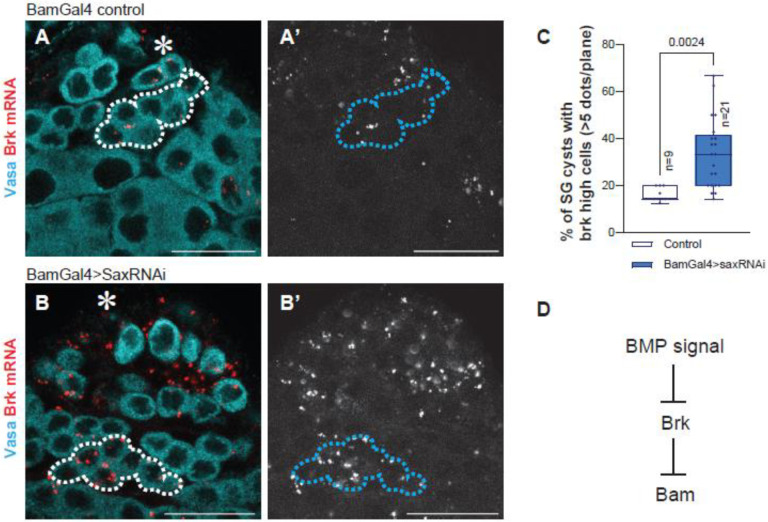
BMP signal suppresses Brk expression in SGs (A, B) Representative testis-tip images of brk mRNA (red) for indicated genotypes. Germ cells are visualized by Vasa staining (Cyan). Broken lines encircle 8-cell SG cysts. (C) The graph shows frequency of the SG cysts with high-brk cells (containing more than 5 brk mRNA). HCR dots were counted from mid-plane cells in SGs. n indicates the number of scored testis. Note that distributions of each SG stage were similar across examined genotypes, indicating that the observed change is not due to SG differentiation defect. p-values were calculated by student-t-test. (D) Suggested model based on the results. Asterisks indicate the hub. All scale bars indicate 20μm.

We first examined the expression pattern of Brk in the testis by in situ hybridization chain reaction (HCR) using previously validated probe against *brk* mRNA^31^. We detected *brk* mRNA in both germline and somatic cells (Figure 3A). *brk* mRNA is absent in GSCs and mainly observed in 4- to −8cell SGs, the stage at which bam expression is normally observed^32^, suggesting the possibility that Brk may contribute to *bam* regulation specifically in Bam positive SGs (Figure 3A).

To test whether BMP signal represses *brk* expression in SGs, we knocked down the typeI receptor Sax, the primary mediator of diffusible BMP signal in SGs^20^. Sax knockdown under the BamGal4 driver led to a significant increase in the number of *brk* transcripts in SGs (Figure 3A–C), indicating that BMP signal negatively regulates *brk* transcription in SGs (Figure 3D).

### Brk is required for maintenance of stem cell pool

In the *Drosophila* testis, GSCs are primarily maintained through asymmetric division, producing one self-renewing GSC and one differentiating daughter cell^11^. However, GSC loss occurs at a certain frequency. Therefore, additional mechanisms are required to sustain GSC population. Our previous study demonstrated that dedifferentiation, whereby displaced daughter cells revert to a GSC identity by re-entering the niche, plays a significant role in maintaining the GSC population^7,33^.

Our previous study has demonstrated that diffusible fraction of Dpp, a major BMP ligand in the niche, promotes SG differentiation by suppressing excess dedifferentiation through activation of *bam* expression^20^. Since Brk represses *bam* expression in SGs, we hypothesized that Brk may be an key factor that induce dedifferentiation via downregulating *bam*. To test this possibility, we examined whether perturbing Brk levels in the germline affects long-term GSC maintenance or not.

**Figure 4.**
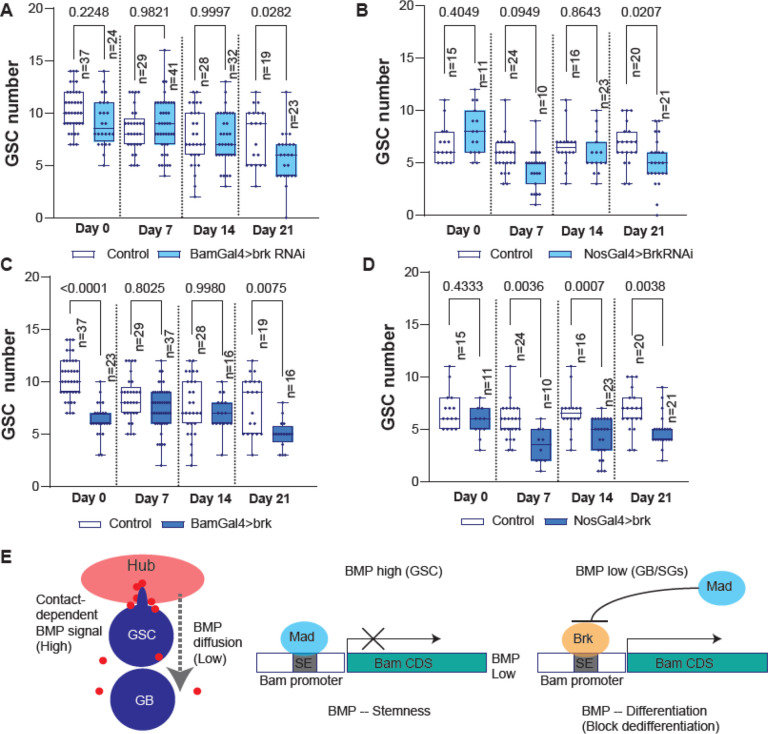
Brk is required for maintenance of stem cell pool (A-D) Changes in GSC numbers during aging in the testes isolated from indicated genotypes. p-values were calculated by Šídák’s multiple comparisons tests in all graphs and provided on each graph. n indicates the number of scored niches (testes). GSC number was scored by confocal imaging of testis tips (see Figure S1 for example). (E) Suggested model based on the results.

Knocking down of Brk under the BamGal4 or NosGal4 driver both resulted in a gradual reduction in GSC number over the fly age, reaching significant by 21 days post-eclosion compared to the control (Figure 4A, B, Figure S1). These effects are reminiscent to the Bam overexpression phenotype and contrary to the Sax knock down phenotype which results in gradual decrease or increase of GSC number shown in previous study^20^, suggesting that Brk removal likely blocks dedifferentiation.

Next, we examined the effect of Brk overexpression. If Brk is an essential factor that induces dedifferentiation, overexpression Brk may accelerate the process and lead to gradual increase of GSC number. However, unexpectedly, we found that overexpression of Brk caused a significant decrease in GSC number throughout all ages via an unknown effect, making it difficult to judge its effect on dedifferentiation (Figure 4C, D). The effect of Brk overexpression on low GSC number is already present in the young flies (day0–7 post-eclosion), suggesting that Brk may play a role in determination GSC number during the niche establishment process independent of its effect on dedifferentiation.

Taken together, these data suggest that the Brk is an essential factor for long term maintenance of GSC number in the niche via regulation of *bam* transcription. The SE integrates both positive and negative regulatory inputs downstream of BMP signaling, with Mad and Brk both serving as repressors that switch on the overlapped binding site in a stage specific manner. Mad is phosphorylated and activated by BMP signal, whereas *brk* expression is suppressed by BMP signal (Figure 4E). This opposed effect of the signal to distinct binding factors may determine whether *bam* remains repressed in GSCs or is upregulated in differentiating progeny.

### Brk gene product displays heterogenous distribution in SG cysts

**Figure 5.**
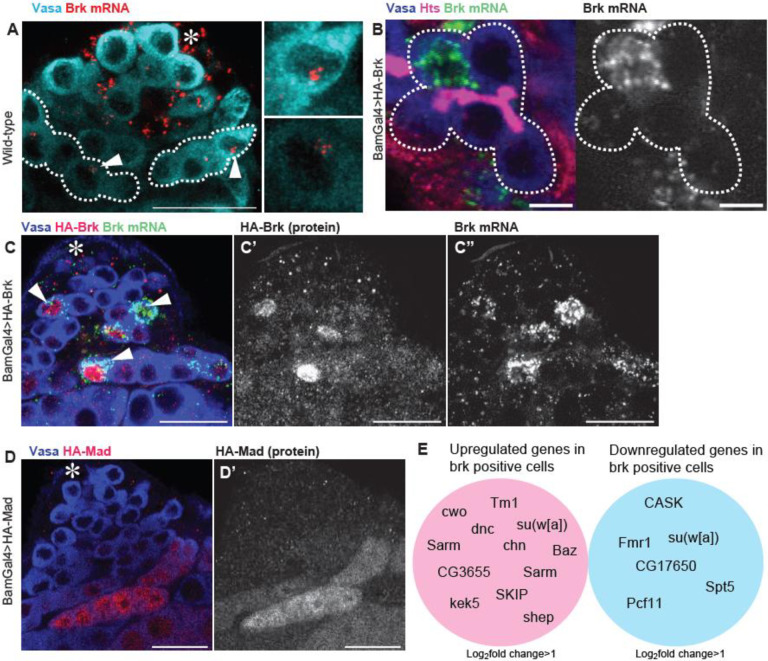
Brk gene product displays heterogenous distribution in SG cysts (A) Representative testis-tip images of brk mRNA (red) in wild type (yw). Germ cells are visualized by Vasa staining (Cyan). Broken lines encircle 8-cell SG cysts. Arrowheads indicate clustered brk mRNA in cells. (B) An example of a 8-cell SG cyst showing biased distribution of overexpressed brk mRNA (BamGal4>HA-Brk). Connection of germ cells is visualized by fusome (Hts staining, red). The broken line encircles a cyst. Right panel shows a brk mRNA channel. (C) Representative testis-tip images of brk mRNA (red) and HA staining in Brk overexpressing testis (BamGal4>HA-Brk). Germ cells are visualized by Vasa staining (blue). Arrowheads indicate Brk highly expressed cells within SG cysts. (D) Representative testis-tip images of HA staining in Mad overexpressing testis (BamGal4>HA-Mad). Germ cells are visualized by Vasa staining (blue). (E) Upregulated and downregulated genes in Brk positive spermatogonia compared with Brk negative spermatogonia based on Fly Cell Atlas, the public platform to analyze single cell RNA sequencing data. Asterisks indicate the hub. Scale bars indicate 5μm for B, 20μm for other images. p-values (non-adjusted) <0.004.

While examining *brk* expression, we observed striking heterogeneity in transcriptional levels of *brk* among cells within individual SG cysts (Figure 5A). In 2–16-cell cysts, *brk* mRNA was predominantly enriched in a small subset of cells, whereas others displayed little or undetectable levels. To determine whether this heterogeneity arises from transcriptional variation or post-transcriptional regulation, we analyzed the distribution of transcripts from a UAS-HA-Brk transgene. As seen in endogenous *brk*, transgene-derived mRNA also accumulated variably in only a small fraction of SG cells, correlating with protein levels detected by HA staining (Figure 5B–C), suggesting that the heterogeneity is acquired via a post-transcriptional regulation. To test whether this phenomenon is specific to Brk, we examined UAS-HA-Mad expression. In contrast to Brk, HA-Mad was detected uniformly across all cells within SG cysts (Figure 5D), suggesting that the variability is a unique feature of the *brk* gene.

The unique heterogeneous expression of Brk prompted us to hypothesize that Brk may prime a subset of SG cells for dedifferentiation. To explore this possibility, we analyzed single-cell RNA-sequencing (scRNA-seq) data using the Fly Cell Atlas platform^34,35^. We extracted SG-clustered cells and compared transcriptomes of Brk-positive versus Brk-negative populations. Intriguingly, *bazooka* (*baz*) emerged as one of the genes significantly enriched in Brk-positive SGs (Figure 5E). Our previous work showed that Baz is enriched in GSCs and required for dedifferentiation^7,14^, raising the possibility of a functional segregation of cells between Brk positive versus negative populations on dedifferentiation.

## Discussion

Using *Drosophila* male gonads, we previously showed that diffusible Dpp plays a critical role outside the niche by preventing excess dedifferention of GSC daughter cells, a function opposite to its niche role of promoting GSC self-renewal. Remarkably, these contrasting outcomes are mediated through the same canonical BMP pathway, which represses *bam* expression in stem cells but upregulates *bam* in differentiating cells^20^. In this study, we identify the transcriptional repressor Brk as a key factor underlying this location-dependent switch in *bam* regulation. Brk is specifically expressed in *bam*-positive populations, where it lowers *bam* transcript levels. At the same time, BMP signaling outside the niche represses *brk* expression, thereby relieving repression and enhancing *bam* expression.

Intriguingly, we identified striking heterogeneity in Brk expression level within SG cysts. Traditionally, cells within *Drosophila* SG cysts are physically interconnected by fusomes, and have been considered equivalent, with all contributing equally to mature gametes^36^. However, our findings reveal, for the first time, an example of molecular heterogeneity within these syncytial cysts, despite their cytoplasmic continuity. Furthermore, analyses of publicly available single cell RNA sequencing data revealed that the Brk-positive SGs may have distinct expression of certain genes. Further study will be necessary to examine the function of these genes and how they affect the behavior of Brk-positive subpopulations.

In summary, our findings indicate that Brk is a critical integrator of diffusible fraction of niche-derived Dpp signals of *bam* in SGs. By acting as a repressor at the *bam* promoter, Brk ensures that *bam* is expressed at appropriate levels to prevent excess dedifferentiation. Whereas it creates a highly heterogenous population within interconnected SG cysts likely to secure cells primed for dedifferentiation. Our work provides the mechanism in which a single niche ligand induces distinct cellular responses inside versus outside the niche, which may be a common mechanism to regulate tissue homeostasis.

## Materials and Methods

### Fly husbandry and strains

Flies were raised on standard Bloomington medium at 25°C, unless temperature control was required. Adult flies (0- to 7-days old) were used in all experiments. The following fly stocks were sourced from the Bloomington Drosophila Stock Center (BDSC): brk RNAi (TRiP.HMC03345, BDSC51789); nosGal4 (BDSC64277); sax RNAi (TRiP.HMJ02118, BDSC42546). For wildtype controls, *yw* (BDSC189) was used. UAS-brk.ORF.3xHA.GW (F000571) UAS-Mad.ORF.3xHA.GW(F001716) were obtained from FlyORF. UASGFP- αTubulin was gifts from Yukiko M. Yamashita.

### Immunofluorescence staining

Testes were dissected in PBS and fixed in 4% paraformaldehyde for 30 min. Samples were then washed in PBST (PBS + 0.2% TritonX-100) for 60 minutes, followed by overnight incubation with primary antibody in PBST containing 3% bovine serum albumin (BSA) at 4°C. Samples were then washed for 60 minutes in PBST, incubated with secondary antibody in 3% BSA in PBST at room temperature for 2 hours and then washed for 60 minutes (three times for 20 minutes each) in PBST. Samples were then mounted using VECTASHIELD with DAPI. The primary antibodies used were as follows: rat anti-Vasa (RRID: AB_760351, 1:20; DSHB); mouse anti-Hts (1B1, 1:20; DSHB) mouse-anti-FasIII (1:20, 7G10; DSHB); Rabbit anti-HA C29F4 (RRID: AB_1549585, 1:300, Cell Signaling Technology, Cat# 3724); AlexaFluor-conjugated secondary antibodies (Abcam) were used at a 1:200 dilution. Images were acquired on a Zeiss LSM800 airyscan using a single z-stack with a 63X/1.4 NA oil objective. Images were processed by image J/FIJI^37^.

### In Situ Hybridization Chain Reaction (HCR)

HCR using a *brk* probe (kind gift from Leslie Dunipace and Angelike Stathopoulos) as performed as described previously^31^. Briefly, testes collected from 3-day-old flies were fixed in 1ml of 4% formaldehyde/PBS for 30 min, then permeabilized in phosphate-buffered saline (PBS) containing 1% Triton X-100 for 2 hours at room temperature, and post-fixed in 4% paraformaldehyde/PBS. Specimens were prehybridized in probe hybridization buffer (Molecular Instruments) for 30 min, followed by overnight hybridization with 0.8 pmol of probe at 37°C. After hybridization, specimens were equilibrated in amplification buffer (Molecular Instruments) for 5 min at room temperature. Hairpin solutions (Molecular Instruments) with 6 pmol of each hairpin were incubated for 90 seconds at 95°C, then cooled down in the dark at room temperature for 30 min. The cooled hairpins were added to the sample in amplification buffer, then incubated overnight (~16 h) at room temperature. Next day, samples were washed with 5x SSC for 5min x2, 30min x1 and 5min x1, then followed by overnight incubation with primary antibody in PBST containing 3% bovine serum albumin (BSA) at 4°C. Samples were then washed for 60 minutes (20 minutes x3) in PBST (PBS + 0.2% TritonX-100), incubated with secondary antibody in 3% BSA in PBST at room temperature for 2 hours and then washed for 60 minutes (three times for 20 minutes each) in PBST. Samples were mounted using VECTASHIELD with 4’,6- diamidino-2-phenylindole (DAPI) (Vector Lab). Images were acquired on a Zeiss LSM800 airyscan using a single z-stack with a 63X/1.4 NA oil objective.

### RNA fluorescence in situ hybridization

Quasar 570 labeled probe against *bam* coding sequence was obtained from LGC Biosearch Technologies. For visualization of germ cells, NosGal4>αTubulin-GFP was used. Testes were dissected in 1X PBS then fixed in 1ml of 4% formaldehyde/PBS for 45 minutes. Fixed testes were rinsed 2 times with 1 ml of 1X PBS, then resuspended in 1ml of ice-cold 70% EtOH, and incubated for 1 hour-overnight at 4 °C. Testes were rinsed briefly in 1 ml of wash buffer (2X SSC and 10% deionized formamide), then incubated overnight at 37 °C for 16 hours in the dark with 50 nM of Stellaris probes in 200 μl of Hybridization Buffer containing 2X SSC, 10% dextran sulfate (MilliporeSigma), 1 μg/μl of yeast tRNA (MilliporeSigma), 2 mM vanadyl ribonucleoside complex (NEB), 0.02% RNase-free BSA (ThermoFisher), and 10% deionized formamide. Then, testes were washed 2 times for 30 minutes each at 37 °C in the dark in 1ml of prewarmed wash buffer (2X SSC, 10% formamide) and resuspended in a drop of VECTASHIELD with DAPI. Images were acquired on a Zeiss LSM800 airyscan using a single z-stack with a 63X/1.4 NA oil objective.

### Quantification of Bam-mGL intensities

The generation of Bam-mGL or Bam(mut)-mGL reporter lines was previously described^20^. To assess the mGL intensity, short-term live imaging was used^7^. Testes from newly eclosed flies were dissected in Schneider’s Drosophila medium supplemented with 10% fetal bovine serum and glutamine–penicillin–streptomycin. Testes were placed on Gold Seal Rite-On Micro Slides with etched rings filled with media and covered with coverslips. Images were acquired on a Zeiss LSM800 airyscan using a single z-stack with a 63X/1.4 NA oil objective. All images were taken using the same acquisition setting. The intensity of Bam-mGL or Bam(mut)-mGL was measured in the 4-, 8-, and 16-cell cyst SG regions. For intensity analysis, signals from each region were measured in ImageJ/Fiji, with background signals (from the same testis) subtracted.

### Single cell RNA seq analysis

Single cell RNA sequencing data were obtained from the Fly Cell Atlas (FlyBase) testis dataset (r_fca_biohub_testis_10x). Raw count data (v2_fca_biohub_testis_10x_raw.h5ad) containing 43,454 cells and 15,695 genes were used to retain the complete gene expression profile including lowly expressed transcriptional regulators.

Cells annotated as “spermatogonium” were filtered from the dataset, yielding 670 cells for analysis. Due to the sparse expression pattern of brk (detected in only 20 out of 670 spermatogonia cells, 3.0%), cells were categorized into two groups based on the binary presence or absence of brk transcripts.

For differential expression analysis, raw count data were normalized to 10,000 counts per cell and log-transformed using scanpy (sc.pp.normalize_total, sc.pp.log1p). Differential expression testing was performed using the Wilcoxon rank-sum test (sc.tl.rank_genes_groups, method=‘wilcoxon’) comparing brk-expressing cells (n=20) versus brk-non-expressing cells (n=650). Full list of differentially expressed genes is provided as Spreadsheet1.

Genes with nominal p-values < 0.004 (uncorrected) were considered for further analysis. It should be noted that no genes reached statistical significance after multiple testing correction (Benjamini-Hochberg, adjusted p-value < 0.05), likely due to the small number of brk-expressing cells and sparse expression patterns typical of single-cell data.

### Statistical analysis and graphing

No statistical methods were used to predetermine sample size. The experiments were not randomized. The investigators were not blinded to allocation during experiments and outcome assessment. Statistical analysis and graphing were performed using GraphPad Prism 10 software. Data are means and standard deviations. All plotted data points are provided in Source Data. Individual numerical values displayed in all graphs are provided in Source data1.

## Supplementary Material

Supplement 1

1
