# Supplementary material for: Brinker regulates reciprocal outcomes of BMP signal between stem cells and differentiating cells
Source: bioRxiv. 2025 Sep 16:2025.09.14.676154. Preprint. [Version 1] doi: 10.1101/2025.09.14.676154 (PMC12458193; doi:10.1101/2025.09.14.676154)
Supplement: Supplementary file 1 [file NIHPP2025.09.14.676154V1-supplement-1.pdf]

bioRxiv preprint doi: https://doi.org/10.1101/2025.09.14.676154; this version posted September 16, 2025. The copyright holder for this preprint (which was not certified by peer review) is the author/funder, who has granted bioRxiv a license to display the preprint in perpetuity. It is made available under aCC-BY 4.0 International license.

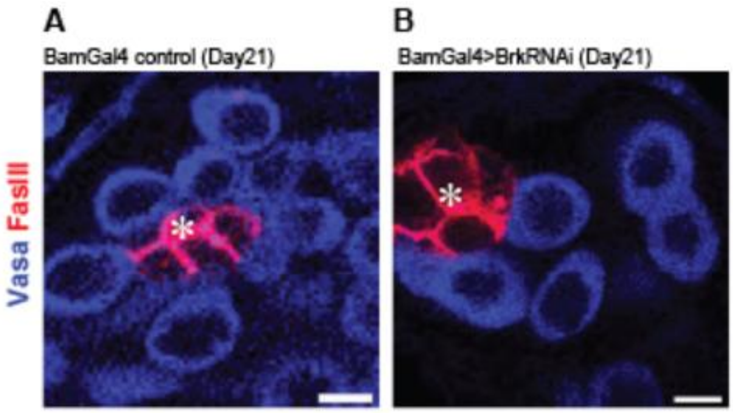

Figure S1. Brk is required for maintenance of stem cell pool

(A, B) Testis-tip images used for GSC number counting for indicated genotypes. The hub is visualized by FasIII (red) staining. Germ cells are visualized by Vasa staining (blue). GSCs were counted as germ cells directly attached to the hub. Imaging was performed to cover entire niche by using z-stacks of 1µm intervals. Asterisks indicate the hub. All scale bars indicate 10µm.
